# Long noncoding RNA LINC01132 enhances immunosuppression and therapy resistance via NRF1/DPP4 axis in hepatocellular carcinoma

**DOI:** 10.1186/s13046-022-02478-z

**Published:** 2022-09-08

**Authors:** Jiwei Zhang, Tao Pan, Weiwei Zhou, Ya Zhang, Gang Xu, Qi Xu, Si Li, Yueying Gao, Zhengtao Wang, Juan Xu, Yongsheng Li

**Affiliations:** 1 Shanghai Key Laboratory of Compound Chinese Medicines, The MOE Key Laboratory for Standardization of Chinese Medicines, Institute of Chinese Materia Medica, Shanghai University of Traditional Chinese Medicine, Shanghai, 201203 China; 2 Key Laboratory of Tropical Translational Medicine of Ministry of Education, College of Biomedical Information and Engineering, Hainan Women and Children’s Medical Center, Hainan Medical University, Haikou, 571199 China; 3 College of Bioinformatics Science and Technology, Harbin Medical University, Harbin, 150081 Heilongjiang China

**Keywords:** Hepatocellular carcinoma, LINC01132, cancer immunotherapy, DPP4

## Abstract

**Background:**

Long noncoding RNAs (lncRNAs) are emerging as critical regulators of gene expression and play fundamental roles in various types of cancer. Current developments in transcriptome analyses unveiled the existence of lncRNAs; however, their functional characterization remains a challenge.

**Methods:**

A bioinformatics screen was performed by integration of multiple omics data in hepatocellular carcinoma (HCC) prioritizing a novel oncogenic lncRNA, LINC01132. Expression of LINC01132 in HCC and control tissues was validated by qRT-PCR. Cell viability and migration activity was examined by MTT and transwell assays. Finally, our results were confirmed in vivo mouse model and ex vivo patient derived tumor xenograft experiments to determine the mechanism of action and explore LINC01132-targeted immunotherapy.

**Results:**

Systematic investigation of lncRNAs genome-wide expression patterns revealed LINC01132 as an oncogene in HCC. LINC01132 is significantly overexpressed in tumor and associated with poor overall survival of HCC patients, which is mainly driven by copy number amplification. Functionally, LINC01132 overexpression promoted cell growth, proliferation, invasion and metastasis in vitro and in vivo. Mechanistically, LINC01132 acts as an oncogenic driver by physically interacting with NRF and enhancing the expression of DPP4. Notably, LINC01132 silencing triggers CD8+ T cells infiltration, and LINC01132 knockdown combined with anti-PDL1 treatment improves antitumor immunity, which may prove a new combination therapy in HCC.

**Conclusions:**

LINC01132 functions as an oncogenic driver that induces HCC development via the NRF1/DPP4 axis. Silencing LINC01132 may enhance the efficacy of anti-PDL1 immunotherapy in HCC patients.

**Supplementary Information:**

The online version contains supplementary material available at 10.1186/s13046-022-02478-z.

## Background

Hepatocellular carcinoma (HCC) is a highly aggressive hepatic malignancy with poor survival rate [1]. The most common therapeutic is surgical resection or liver transplantation [2]. However, patients are usually diagnosed at an advanced stage and are not suitable for surgical treatment. Therefore, systematic investigation of the underlying mechanism associated with HCC development and progression is of high clinical significance and may lead the development of novel clinical options [3].

With the development of high-throughput sequencing technologies, numerous molecular markers have been linked to the development of HCC. Genes from various signaling pathways are frequently mutated in HCC, such as Wnt, P53, AKT and MAP kinase pathways [4]. Copy number alterations have been found to be involved in the development of HCC, such as MET and PEG10 amplification [5, 6]. Cancer-related mutations have been found to perturb the RNA regulatory network and helped the identification of potential biomarkers [7]. However, the molecular pathogenesis of HCC is still not fully understood, and novel cancer-promoting genes must be identified and characterized.

Current progress in transcriptome analysis exposed a large portion of the human genome that does not encode for proteins [8]. Long noncoding RNAs (lncRNAs) have been discovered as a major type of regulatory RNA with important roles in cancer development [9]. Accumulating evidence has shown that lncRNAs are involved in a wide range of biological processes, acting as scaffolds or miRNA sponges [10, 11]. For example, LINC01138 has been found to drive malignancies via activating arginine methyltransferase 5 in HCC [12]; to physically interact with the MYC protein and increase its stability in cancer [13]; and inflammation induced LINC00665 is involved in the NF-kB signaling activation in HCC [14]. Many lncRNAs were identified as novel regulatory RNAs in HCC but their biological functions and underlying mechanism in pathogenesis remain largely unclear.

The crosstalk between the immune system and tumor cells is critical in cancer development and progression [15]. Following the success story of immune checkpoint blockers (ICBs) therapy in various cancer types, increasing efforts were devoted to investigate novel ICBs approaches in HCC patients [16]. Despite this breakthrough, a subset of patients remains as non- ideal candidates for immunotherapy due to the lack of efficacy [17, 18]. Therefore, numerous studies focused on modifying the expression of noncoding RNAs in combination with immunotherapy to improve the response and overall survival. LIMIT is an immunogenic lncRNA in cancer immunity targetable for cancer immunotherapy [19], while NKILA, another lncRNA, can promote tumor immune evasion by sensitizing T cells and activate induced cell death [20]. Furthermore, pan-cancer analysis of immune-related lncRNAs has prioritized cancer-related lncRNAs and the identification of immune subtypes [21]. Therefore, exploring the roles of lncRNAs in immune regulation is essential to identify additional immunotherapy targets in cancer.

In this study, we identified a new oncogenic long intergenic noncoding RNA (lincRNA) in HCC (LINC01132). LINC01132 is overexpressed in HCC, and significantly associated with malignant clinical features and poor outcomes in the clinic. Mechanistically, LINC01132 promotes cell growth, proliferation, invasion and metastasis through the LINC0132/NRF1/DPP4 axis. Finally, combinatory therapy targeting LINC01132 inhibition and anti-PDL1 blockade synergistically improves antitumor immunity in HCC in vivo and ex vivo models. In summary, our data suggests that LINC01132 is a potential biomarker and therapeutic target for HCC.

## Materials and methods

### Patients and ethical statement

In total, 121 HCC patients’ tumor samples and corresponding adjacent normal liver tissues were obtained from the surgical specimen archives of the Zhongshan Hospital [12], Shanghai, China. The patients were informed, and signed consent forms acknowledging the use of their resected tissues for research purposes, which has been previously approved [12].

### Cell culture

The HCC cell lines HepG2 and Hep1–6 were purchased from American Type Culture Collection (ATCC, Manassas, Virginia, USA) and cultured following the recommended guidelines. These cells were characterized by Genewiz Inc. cultured in Dulbecco’s Modified Eagle’s Medium (DMEM) (Thermo Fisher Scientific, California, USA) with 10% fetal bovine serum (FBS) and antibiotics. Huh-7 cells were was purchased from JCRB cell bank (Tokoyo, Japan) and cultured in Roswell Park Memorial Institute (RPMI) 1640 Medium (Thermo Fisher Scientific) with 10% new born calf serum. Hep3B and SUN-449 were purchased from the Cell Bank of Chinese Academy of Sciences (Shanghai, China). Cells were maintained in Dulbecco’s modified eagle medium (DMEM, Invitrogen, Carlsbad, CA, USA) supplemented with 10% fetal bovine serum (FBS, HyClone, Logan, UT, USA), 1% penicillin/streptomycin (pen/strep, Invitrogen), and 8 mg/L of the antibiotic, tylosin tartrate, for mycoplasma (Sigma-Aldrich, St. Louis, Missouri, USA), at 37 °C in 5% CO2 (v/v). All cell lines were authenticated by autosomal STR profiling and thawed afresh every 2 months, to test for mycoplasma. None of the cell lines used was found in the database of commonly misidentified cell lines, maintained by the International Cell Line Authentication Committee.

### In vivo mouse models

All studies were supervised and approved by the Shanghai University of Traditional Chinese Medicine Institutional Animal Care and Use Committee (IACUC). Female mice were used as models to study liver cancer. Power analysis indicated an *n* value of 5 mice per group to identify the expected effects with 90% confidence.

### RNA quantization

Total RNA was extracted from liver samples or HepG2 or Huh-7 cells with TRIzol Reagent (Thermo Fisher Scientific, California, USA). Quantitative real-time PCR (qPCR) was performed with an iQ5 machine and SYBR Premix Ex Taq™ II (TaKaRa Bio, Tokyo, Japan). Data were normalized to β-actin or IgG control (RNA pull-down assay and RIP assay). Relative genomic level of tumor tissues was compared with normal liver tissue. Primers used for RT-qPCR and RT-PCR are described in additional file 1: Table S1.

### Cytoplasmic and nuclear RNA isolation

Cytoplasmic and nuclear RNA was extracted using Thermo Fisher BioReagents (Thermo Fisher Scientific) according to the manufacturer’s instructions. QRT-PCR analysis was performed using SYBR® Green Master Mix (Invitrogen, New York, USA) to amplify the localization of LINC01132 assay and β-actin U6 were used as cytoplasmic and nucleus controls. Primers are listed in additional file 1: Table S1.

### Lentivirus construction and infection

The LINC01132 sequence (listed in additional file 1: Table S1) was amplified from normal genomic RNA and cloned into the pWPXL lentiviral vector to generate pWPXL-LINC01132. Virus particles were harvested 48 hrs after HEK 293 T cells were transfected with pWPXL-LINC01132, with the packaging plasmid psPAX2 and the VSV-G envelope plasmid pMD2.G using Lipofectamine 2000 reagent (Invitrogen). Huh-7, HepG2, Hep3B, SNU-449 and Hep1–6, cells were infected with recombinant lentivirus-transducing units in the presence of 1 μg/ml polybrene (Sigma-Aldrich, Missouri, USA).

### LINC01132 adenovirus interference vector (AD-LINC01132-SHRNA)

Three pairs of LINC01132-shRNA oligonucleotide sequences were designed and three adv4-U6-CMV-LINC01132-shRNA adenovirus shuttle plasmids were constructed. The constructed ADV4-U6-CMV-LINC01132-shRNA and pHBAd-BHG plasmids were co-transfected into 293A cells and packaged with AD-LINC01132-shRNA. The virus titer was detected by microcytic whole-cell assay. Ad-linc01132-shrna was injected subcutaneously and the mRNA expression of LINC01132 was detected by real-time fluorescence quantitative PCR.

### In vitro cell proliferation and colony formation assays

The cell proliferation assay was measured using the Cell Counting Kit-8 (CCK-8) (Dojindo, Kyushu, Japan) at 1, 3, and 5 days after LINC01132 or mock infection. Cells were seeded into the 96-well plate at a density of 10^3^ cells/well, and 10 μl of CCK-8 was added to 90 μl of the cell culture medium per well. Cells were subsequently incubated at 37 °C for 2 hours and the optical density measured at 450 nm. For the colony formation assay, 1–1.5 *10^3^ cells/well were plated in a 6-well plate and incubated at 37 °C for 2 weeks. The colonies were fixed and stained with 0.1% crystal violet dye in 20% methanol, and the number of colonies macroscopically counted. All assays were performed in triplicate.

### In vitro migration and invasion assays

Migration assays were performed in a Transwell chemotaxis 24-well chamber (BD Biosciences, Franklin Lakes, NJ). Briefly, 2 × 10^4^ cells were plated in the upper chamber with a non-coated membrane. For the invasion assays, 5 × 10^4^ cells were placed into the upper chamber with a Matrigel-coated membrane. After 16 hours of incubation at 37 °C, migrating or invading cells were fixed and stained with 0.1% crystal violet dye in 20% methanol. Migrated or invaded cells were counted and imaged with an inverted microscope (Olympus, Tokyo, Japan).

### In vivo assays for metastasis

For the in vivo metastasis assays, 2 × 10^6^ Hep1–6 cells infected with the pWPXL-LINC01132 or pWPXL-GFP were resuspended in 0.2 mL of serum-free DMEM and subcutaneously injected into C57BL/6 mice liver. After 40 days mice humanly euthanized and liver, lungs and intestines were collected, fixed with phosphate buffered neutral formalin and prepared for standard histological examination. The numbers of metastatic foci in liver tissue sections were counted by H&E staining under a binocular microscope (Leica, USA).

### Construction of PDX mouse model of HCC

PDX model of liver cancer was established from patient’s liver cancer samples. These were first sectioned into small tissue for subcutaneous tumor formation in nude mice. Two weeks later, the subcutaneous tumor bearing tissue was excised, sectioned and and transplanted again into the subcutaneous skin of nude mice. After subcutaneous tumor formation, shNC and shLINC01132 were injected subcutaneously and tumor diameter and weight were measured every 2 days. Animals were humanly euthanized after 30 days and tumor tissue removed for terminal endpoint analysis.

### RNA pull-down assays

LINC01132 was transcribed in vitro with biotin RNA labelling mix and T7 RNA polymerase according to the manufacturer’s instructions (Invitrogen). In total, 40 μl streptavidin-linked magnetic beads (ThermoFisher Scientific) were used to pull down the biotinylated RNA at room temperature for 2 hrs. The beads-RNA-proteins were then washed with 1× binding washing buffer (5 mM Tris-HCl, 1 M NaCl, 0.5 mM EDTA, and 0.005% Tween 20) four times. The proteins were precipitated and diluted in 60 μl protein lysis buffer. Finally, the retrieved proteins were measured on SDS-PAGE gels for mass spectrometry or Western blot. Western blot in RNA pull-down assay was performed with mouse anti-NRF1 and anti-KDM5B antibodies (Cell Signaling Technology, CST, Danvers, Massachusetts, USA, 1:500) and mouse anti-β-actin antibody (CST, 1:1000). Antibodies information is available on additional file 1: Table S2.

### RNA-seq and computational analysis

RNA-seq was performed at the Sequencing and Non-Coding RNA Program at the RiboBio (Guangzhou, China) using Hiseq3000(Illumina, USA). The hisat2 [22], StringTie [23] and Ballgown were used to align the reads to the genome, generate raw counts corresponding to each known gene, and calculate the RPKM (reads per kilobase per million) values [24].

### Northern blot

LINC01132 levels were measured by northern blot using an Ambion Northern Max-Gly Kit (Austin, TX, USA). Total RNA was electrophoresed and siphoned to a positively charged nylon membrane (NC). RNA was then fixed to the NC membrane using UV cross-linking. In brief, the cross-linked membrane was then prehybridized with ULTRAhyb, and RNA was detected with an LINC01132-specific oligonucleotide probe (primers provided in additional file 1: Table S1) labeled with digoxigenin-ddUTP using a DIG Oligonucleotide 3′-End Labeling Kit (Roche Diagnostics, Indianapolis, IN, USA) in roller bottles.

### Western blot

Proteins were separated by sodium dodecyl sulfate-polyacrylamide gel electrophoresis (SDS-PAGE) and transferred to nitrocellulose membrane (Bio-Rad, Hercules, CA). Non-specific binding was blocked with 5% nonfat milk and subsequently incubated with indicated primary antibodies, followed by horseradish peroxidase-conjugated secondary antibodies. Immunoreactivity was visualized with chemiluminescence ECL reagents (Pierce, Rockford, IL) and imaged with ChemiDoc imaging system (12003153-s). Densitometry analysis was performed with Image-Pro Plus 6.0 (Media Cybernetics).

### Mass spectrometry analysis

Specific bands were excised for proteomics screening by mass spectrometry analysis (Shanghai Applied Protein Technology, Shanghai, China). Protein identification was retrieved from the human RefSeq protein database (National Center for Biotechnology Information), using Mascot version 2.4.01 (Matrix Science, London, UK). The retrieved protein was detected by western blot.

### RNA immunoprecipitation (RIP)

RIP experiments were performed using the Magna RIP™ RNA-Binding Protein Immunoprecipitation Kit (Millipore, Massachusetts, USA) according to the manufacturer’s instructions. The co-precipitated RNAs were detected by reverse transcription PCR. Total RNA (input controls) and normal mouse IgG controls were assayed simultaneously to validate RNA specificity to NRF1 and KDM5B (*n* = 3 for each experiment). Gene-specific primers for LINC01132 are provided in additional file 1: Table S1).

### Co-immunoprecipitation

Huh-7 and HepG2 cells infected with the lentivirus expressing LINC01132 or Huh-7 cells transfected with LINC01132 siRNA were lysed with RIPA buffer (Beyotime Biotechnology) with protease (Thermo Fisher Scientific Inc.) and RNase (Thermo Fisher Scientific Inc.) inhibitors, and then centrifuged at 16,400 g for 15 min. Supernatants were collected and the amount of NRF1 and DPP4 or KDM5B and DPP4 protein examined by immunoblotting to normalized for DPP4 and NRF1 loading. Supernatants were then incubated with the indicated antibody-coated Protein G Dynabeads (Life Technologies) overnight at 4 °C with gentle rotation. The beads were washed five times with NT2 buffer (50 mM Tris-HCL [pH 7.4], 150 mM NaCl, 1 mM MgCl2, 0.05% Nonidet P-40) containing protease and RNase Inhibitor, and then three times with PBS again containing protease and RNase Inhibitor. After washing, proteins were eluted and immuno-complexes were analyzed by immunoblotting (antibodies provided in additional file 1: Table S2).

### Genome-wide expression of lncRNAs in HCC

Genome-wide expression of lncRNAs in HCC was collected from Gene Expression Omnibus (GEO) and The Cancer Genome Atlas (TCGA). Gene expression profile of HCC was collected from GEO under the accession number GSE104310, which provided the expression of tumors and paired non-tumor tissues collected from Sun Yat-sen University Cancer Center. In total, eight normal and 12 tumor samples were included in our analysis. Moreover, genome-wide expression of liver hepatocellular carcinoma (LIHC) were obtained from TCGA project [25], which included 374 tumor and 50 normal samples. RNA sequencing of 21 Hepatitis B virus-HCC patients with non-neoplastic liver and tumor tissues were obtained from GSE94660 [26].

### Identification of differentially expressed lncRNAs in HCC

Wilcoxon’s rank sum test was used to identify differentially expressed lncRNAs in HCC tumor compared to normal tissues. Only long intergenic non-coding RNAs (lincRNAs) were considered in our analyses. We identified the lincRNAs with fold changes > 2 or < 0.5 and adjusted *p*-values < 0.05 as differentially expressed lincRNAs.

### Predict the functions of lncRNA

Spearman correlation coefficients (SCC) were used to predict the function of lncRNA and protein-coding gene expression. All genes were ranked based on SCC and subjected into Gene Set Enrichment Analysis (GSEA) [27]. The Biocarta pathway dataset from MsigDB was used for this analysis [28]. Pathways with normalized enrichment score > 1.96 or < − 1.96 and false discovery rate (FDR) < 0.01 were identified.

### Transcription regulation analysis

To identify the transcription factors (TFs) that bind to the promoter region of gene (e.g., DPP4), we queried the ChIPBase database [29]. The TFs than can bind to the 5 kb upstream to 1 kb downstream of transcript start sites were identified.

## Results

### Integrative analyses identify onco-lncRNA LINC01132 in HCC

**Fig. 1:**
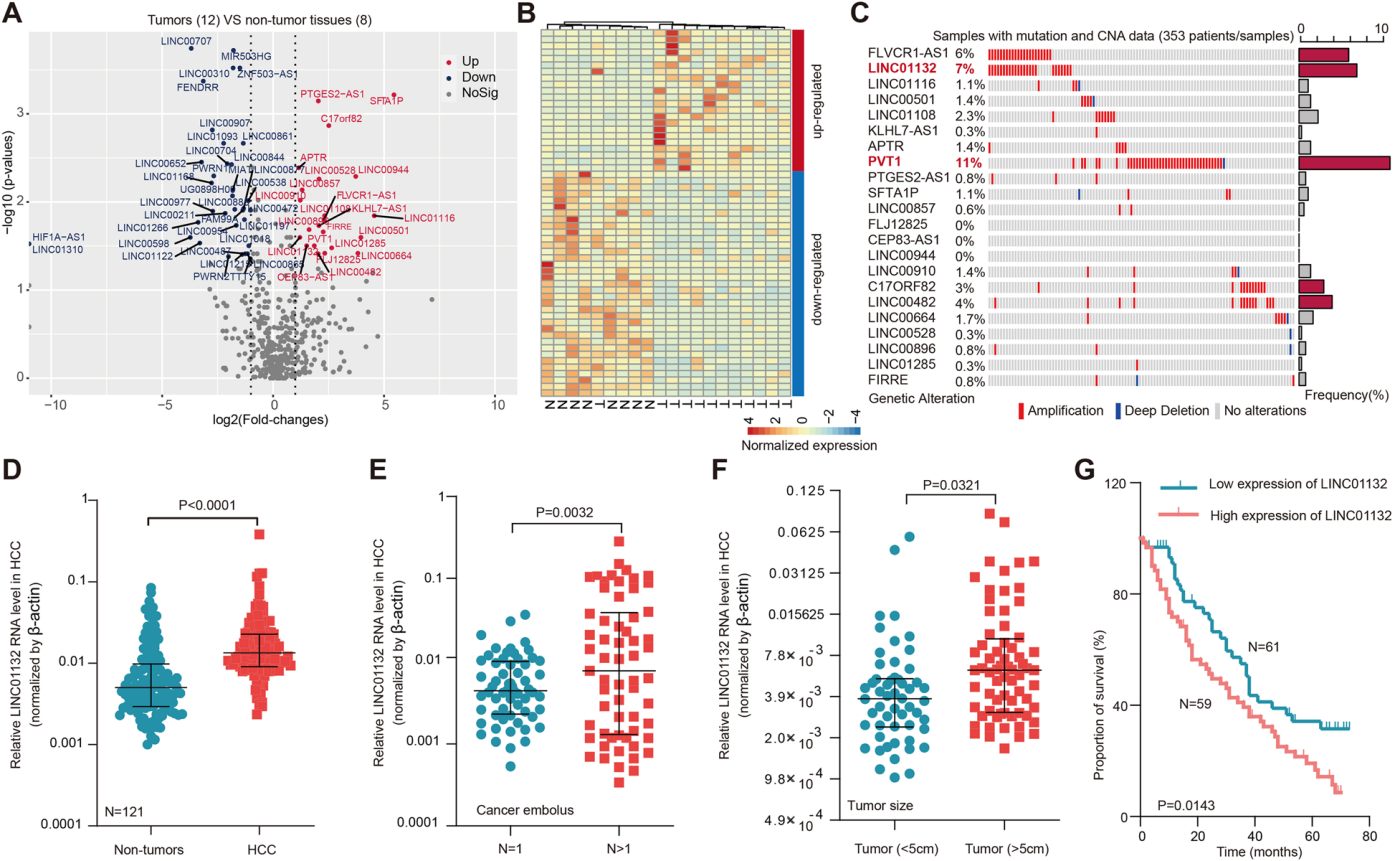
Integrative analyses of multidimensional omics data revealed candidate lincRNAs in HCC. **A** Volcano plot showing the differentially expressed lincRNAs in HCC. Red, up-regulated lincRNAs; blue, down-regulated lincRNAs. **B** Heat map showing the expression of lincRNAs across tumor and normal samples. **C** Waterfall plot showing the somatic mutations and copy number alterations of up-regualted lincRNAs in HCC. The barplot on the right showing the altered frequency. **D** Boxplots showing the expression of LINC01132 in paired tumor and normal samples. Red, HCC tumors and blue, normal controls. **E** Boxplots showing the expression of LINC01132 in tumor samples with one cancer embolus vs. > 1 embolus. **F** Boxplots showing the expression of LINC01132 with different tumor sizes. **G** Kaplan-Meier survival analysis of HCC patients stratified by the LINC01132 expression level

To identify the potential oncogenic lncRNAs in HCC, we first analyzed the genome-wide expression profile of 12 tumors and 8 adjacent non-tumor tissues. In total, we identified 22 up-regulated long intergenic noncoding RNAs (lincRNAs) and 35 down-regulated lincRNAs in HCC (Fig. 1A and additional file 1: Table S3). Numerous tumor associated lincRNAs were identified, such as PVT1 [30], HIF1A-AS1 [31] and MIAT [32]. Moreover, the expression of these lincRNAs can effectively distinguish the tumor tissue from the normal controls in HCC (Fig. 1B).

Next, we investigated the genetic alterations of differentially expressed lincRNAs. The majority of highly expressed lincRNAs were associated with copy number amplification in HCC (Fig. 1C). For example, approximate 11% of HCC patients had PVT1 CNV amplification, which might induce the expression of PVT1 in HCC. In the contrast, lower number of patients had copy number deletion for the down-regulated lincRNAs (Additional file 2: Fig. S1A). We then focused on the lincRNA-LINC01132, which exhibited the second higher copy number amplification (7%) in HCC (Fig. 1C) and was highly expressed in HCC tissue. Moreover, LINC01132 exhibited higher copy number amplification across various cancer types (Additional file 2: Fig. S1B).

To further validate the oncogenic roles of LINC01132, we analyzed the expression in another two cohorts of HCC. We found that LINC01132 exhibited significantly higher expression in tumor patients (Additional file 2: Fig. S1C-D). We also evaluated the expression of LINC01132 in 121 paired tumor tissues and corresponding non-cancerous tissues (NCT), showing that LINC01132 was highly expressed in cancer (Fig. 1D, *p* < 0.0001). Patients with more than one cancer embolus (Fig. 1E, *p* = 0.0032) or larger tumor size (Fig. 1F, *p* = 0.0321) had even higher LINC01132 expression levels. Finally, we explored the effects of LINC01132 on prognosis in HCC patients and observed that high LINC01132 expression was associated with poor survival of HCC (Fig. 1G, *p* = 0.0143). Altogether, these results suggested that LINC01132 plays an oncogenic role in HCC.

### LINC01132 promotes HCC cell growth and metastasis in vitro

To investigate the possible roles of LINC01132 in HCC pathogenesis, we first explored the expression in HCC cell lines. We found that LINC01132 was with relatively high expressed in HepG2 and but not in Huh-7 (Additional file 2: Fig. S2A). We also observed that the expression of LINC01132 was in both cytoplasm and nuclear (Additional file 2: Fig. S2B), suggesting that it may play a regulatory function in both cellular localizations. Moreover, we found that LINC01132 didn’t express to protein but functioned as RNA (Additional file 2: Fig. S2C-D).

**Fig. 2:**
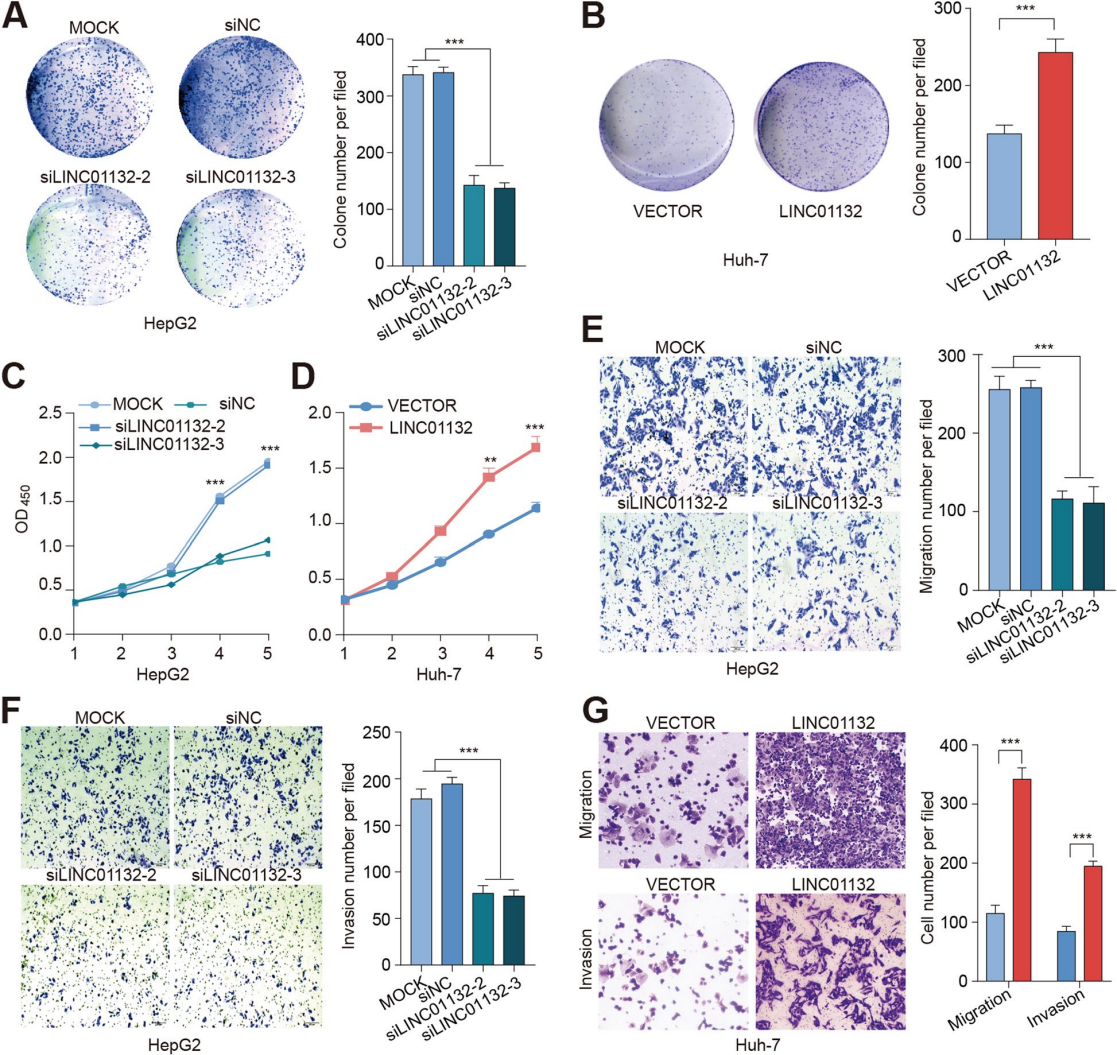
LINC01132 increases cancer cell growth, proliferation, invasion and metastasis in vitro. **A** Colony formation assays of the effects of siLINC01132 vs. controls. **B** Colony formation assays of the effects of overexpression of LINC01132 vs. controls. **C-D** Cell Counting Kit-8 (CCK-8) assays showing the growth of HCC cells treated with siLINC01132 (**C**) or overexpression of LINC01132 (**D**). **E**-**G** Transwell migration and invasion assays in HCC cell lines treated with siLINC01132 or overexpression of LINC01132. **E** for migration treated with siLINC01132, **F** for invasion treated with siLINC01132 and **G** for migration and invasion treated with overexpression of LINC01132

We next knocked down or overexpressed LINC01132 in HCC cell lines and performed colony and cell proliferation assays. We found that the knocked down or overexpressed LINC01132 can effectively alter its expression in cell lines (Additional file 2: Fig. S2E). LINC01132 knocked down significantly decreased the number of colony formation (Fig. 2A, *p* < 0.001) while overexpression of LINC01132 significantly increased the number of colony formation (Fig. 2B, *p* < 0.001). Moreover, knockdown of LINC01132 significantly decreased cell proliferation (Fig. 2C, *p* < 0.001), while LINC01132 overexpression significantly promoted the cancer cells proliferation (Fig. 2D, *p* < 0.001).

We next explored the roles of LINC01132 in cell invasion and migration. LINC01132 knockdown significantly decreased cell migration and invasion (Fig. 2E-F, *p*-values < 0.001). In the contrast, overexpression of LINC01132 significantly increased cell migration and invasion (Fig. 2G, *p*-values < 0.001). In addition, we validated the biological roles of LINC01132 in another two cell lines (Hep3B and SNU-449). We found that the results were consistent across different cell lines (Additional file 2: Fig. S3). Taken together, these results demonstrated that LINC01132 significantly promotes in vitro cell growth, proliferation, migration and invasion in HCC.

### LINC01132 promotes HCC cell growth and metastasis in vivo

**Fig. 3:**
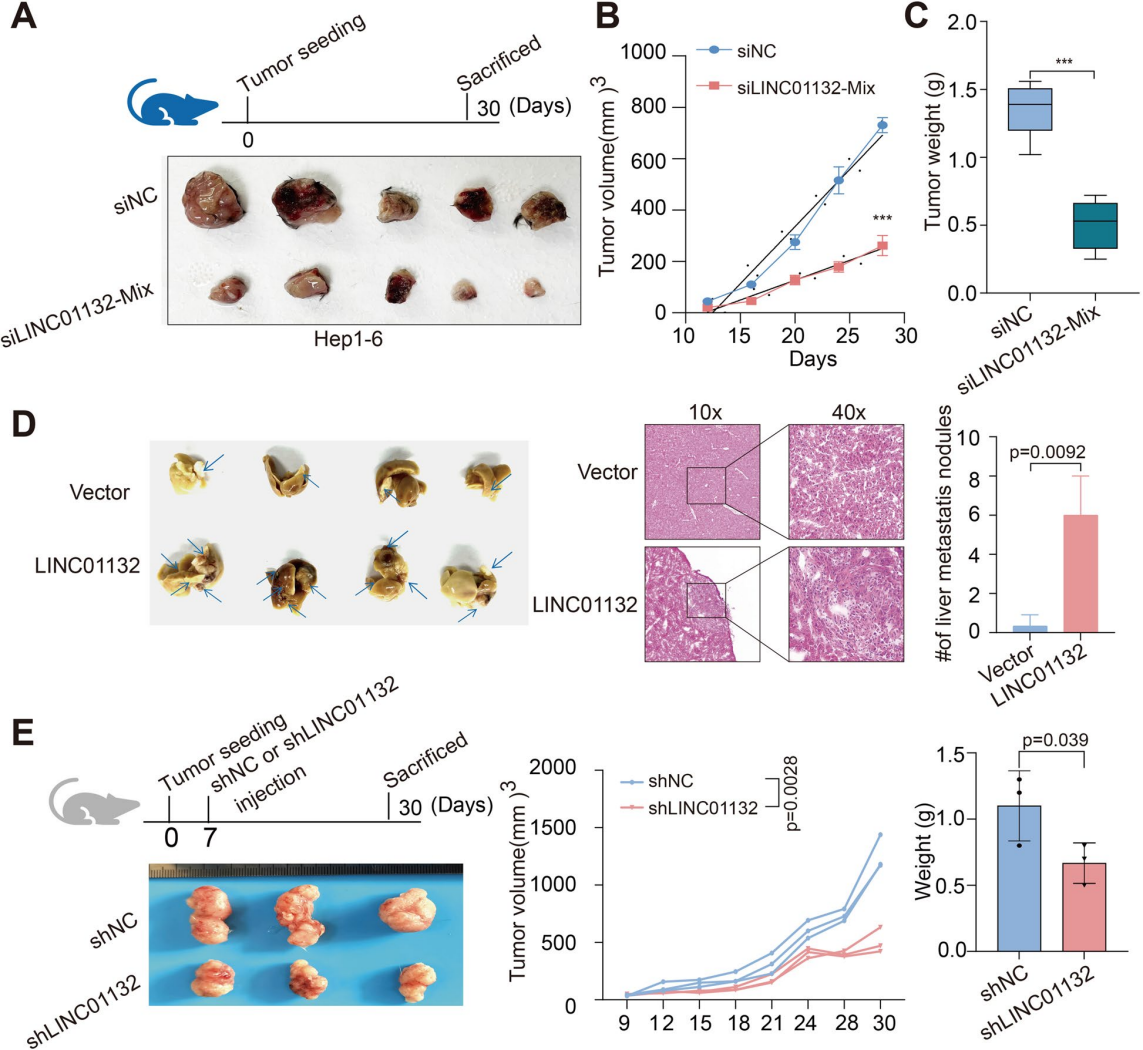
LINC01132 promotes tumor growth and metastasis in vivo. **A** The tumor volume of Hep 1–6 control and siLINC01132 in C57BL/6 mice. **B** The lines showing the tumor volume of Hep 1–6 control and siLINC01132 in C57BL/6 mice at different days. **C** Boxplots showing the tumor weights of Hep 1–6 control and siLINC01132 in C57BL/6 mice. **D** The histogram showing the numbers of liver metastasis nodules in control and overexpression of LINC01132. Top representative pictures of transwell assay. **E** PDX models shLINC01132. The lines showing the tumor volumes of PDXs. The histogram showing the tumor weights of PDXs

To confirm the functions of LINC01132 on the tumorigenicity of HCC, LINC01132 knockdown cells and control cells derived from cell lines were subcutaneously injected into nude immunodeficient mice. Tumor xenografts derived from LINC01132-knockdown cells exhibited smaller volumes and lower weights than those from empty vector-transduced cells (Fig. 3A-C). In addition, we assessed the effects of LINC01132 on metastasis, observing that the numbers of metastatic nodules were significantly increased in LINC01132-overexpressing groups (Fig. 3D, *p* = 0.0092).

The patient-derived xenograft (PDX) mouse model has been shown to recapitulate multiple characteristics of human cancer biological context. Therefore, we next silenced LINC01132 on PDX mouse model 7 days post inoculation (Fig. 3E). LINC01132 knockdown significantly inhibited cancer growth as shown by decreased tumor volumes and weights (Fig. 3E, *p* = 0.0028 and 0.039). As the transfection efficiency of plasmid vector was low, and adenovirus vector was constructed to validate the function of LINC01132. We found that silence of LINC01132 induced significantly decreased tumor volumes and weights (Additional file 2: Fig. S4). Together, these results indicated that elevated LINC01132 expression may contribute to the development and progression of HCC.

### LINC01132 potentially regulates DPP4 in HCC

**Fig. 4:**
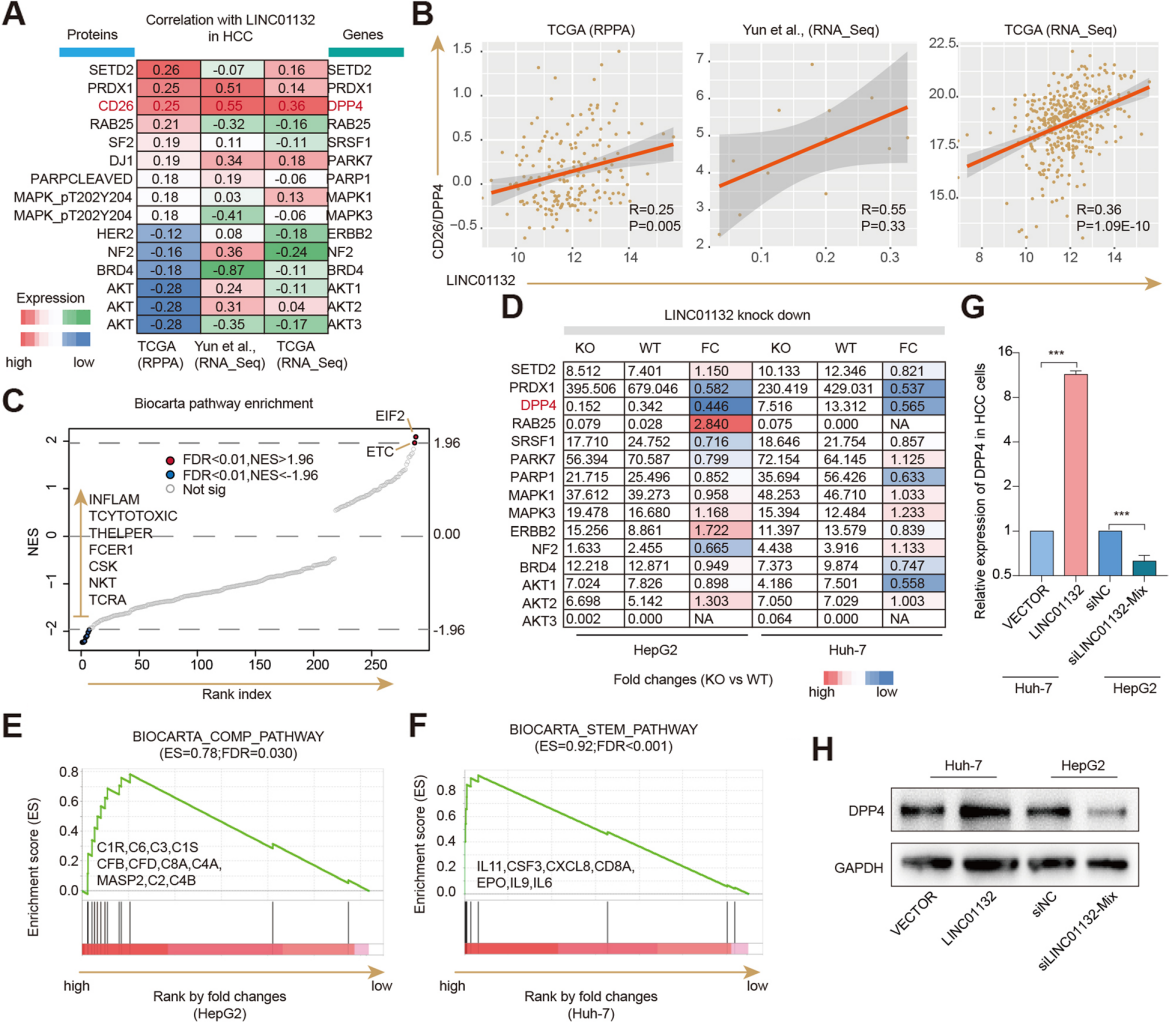
LINC01132 potentially regulates immune-related pathways via DPP4 in HCC. **A** Heat map showing RNAs and proteins correlated with LINC01132 in HCC. The first column represents the correlation based on protein expression. The second and third columns represent the correlation based on RNA expression. **B** Scatter plots showing the correlation between LINC01132 expression and CD26 (DPP4) expression in HCC. **C** Biocarta pathways enriched by LINC01132-correlated genes. Pathways were ranked based on the normalized enrichment scores (NESs). Significantly positively and negatively correlated pathways were indicated in the plot. **D** Heatmap showing the expression and fold changes of genes after knock down of LINC01132 in HCC cell lines. **E-F** Enrichment plots of COMP and STEM pathways identified by genes coexpressed with LINC01132 in HCC. **E** for COMP pathway and **F** for STEM pathway. **G** Relative RNA expression of DPP4 in cell lines treated with knock down and overexpression of LINC01132. **H** Relative protein expression of DPP4 in cell lines treated with knock down and overexpression of LINC01132

To elucidate the potential molecular mechanisms of LINC01132 in HCC, we calculated the correlation between protein expression and expression of LINC01132 in TCGA HCC cohort [33]. LINC01132 expression was significantly positively correlated with SETD2, PRDX1 and CD26, while negatively correlated with AKT and BRD4 (Fig. 4A). Deficiency of histone methyltransferase SET Domain-Containing 2 (SETD2) in liver has been demonstrated to lead to abnormal lipid metabolism and HCC [34]. Similarly, PRDX1 can act as a pro-cancer protein in HCC HepG2 cells [35]. The expression of LINC01132 was consistently correlated with CD26 protein and RNA expression in HCC (Fig. 4B). CD26/dipeptidyl peptidase (DPP) 4 is a membrane-bound protein found in many cell types and has been suggested as a potential biomarker and target for cancer therapy [36].

Next, we performed GSEA analysis based on the expression correlation between LINC01132 and all protein coding genes and found that LINC01132 expression was positively correlated with EIF2 and ETC pathways, while negatively correlated with TCRA and NKT pathways (Fig. 4C). EIF2 pathway has been associated with various types of cancer, including HCC [37, 38]. The observed negative correlation with immune-related pathways suggests that LINC01132 might suppress immune pathways to promote the cancer development and progression.

Moreover, we in two cell lines and performed RNA-Seq for genome-wide expression profiling. Potential correlated genes such as PRDX1, DPP4 (CD26) and AKT2 exhibited significant expression changes in, LINC01132 knockdown cells (Fig. 4D). GSEA analysis revealed that highly expressed genes LINC01132 knockdown cells were significantly involved in COMP and STEM pathways (Fig. 4E-F, false discovery rates = 0.030 and < 0.001). In particular, we found that LINC01132 knockdown increased the expression of numerous immune-related genes, such as CD8A, IL6 and IL11 (Fig. 4F). We further investigated the expression levels of DPP4 in LINC01132-knockdown or LINC01132-overexpressing HCC cells. DPP4 transcriptional and protein levels were increased LINC01132-overexpressing and decreased in LINC01132-knockdown HCC cells, respectively (Fig. 4G-H). These results suggest that LINC01132 might exert its oncogene functions by modulating the DPP4 signaling pathway.

### LINC01132 interferes with NRF1 binding to DPP4 in HCC

**Fig. 5:**
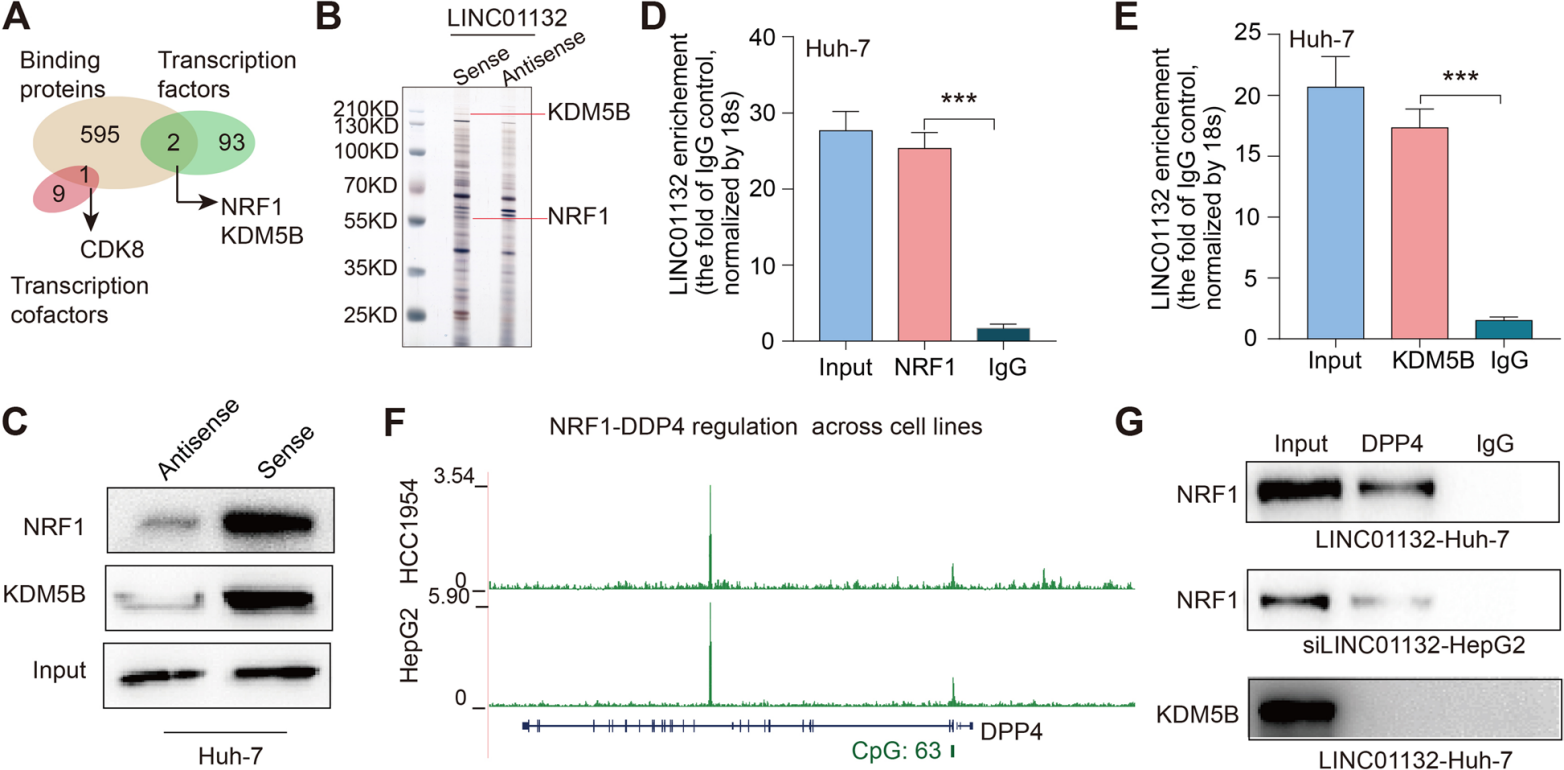
NRF1-DDR4 regulatory axis in HCC. **A** Venn plot showing the overlap of LINC01132 binding proteins and transcription factors, cofactors binding to DPP4 promoter region. **B** SDS-PAGE staining results of LINC01132 pull down assay. **C** Immunoblotting for the specific associations of NRF1 or KDM5B with biotinylated-LINC01132 from streptavidin RNA pull-down assays. **D**-**E** RIP assays were performed using the indicated antibodies. Real-time PCR was used to detect LINC01132 enrichment, using IgG antibody as the control. **D** for NRF1 and **E** for KDM5B. **F** Integrative Genomics Viewer of NRF1 binding around the TSS of DPP4 in HCC cell lines. **G** Immunoblotting of NRF1 and KDM5B in samples from DPP4 RIP assays with LINC01132 overexpression and knock down

To further explore the molecular mechanism underlying the oncogenic activity of LINC01132 in HCC, we performed RNA pull-down assay to identify the proteins associated with LINC01132. LINC01132 pull-down identified 598 potential interacting proteins (Fig. 5A). Of these proteins, NRF1 and KDM5B, and one transcription cofactor (CDK8) have binding sites around the transcription start site (TSS) of DPP4 gene (Fig. 5A). Moreover, we confirmed that sense but not antisense LINC01132 was specifically associated with NRF1 and KDM5B (Fig. 5B-C). RIP assays further showed that NRF1 and KDM5B antibodies could significantly enrich LINC01132 whereas the GAPDH antibody and IgG control could not (Fig. 5D-E). The expression of NRF1 and KDM5B was then investigated showing that higher grade HCC patients exhibited significantly higher expression of NRF1 (Additional file 2: Fig. S5A-B). Survival analysis revealed that poor survival of patients with higher expression of NRF1, KDM5B, LINC01132 and DPP4 (log-rank *p* = 0.038, Additional file 2: Fig. S5C).

Furthermore, we retrieved the public chromatin immunoprecipitation (ChIP) sequencing data from ChIPBase [29] and found that the NRF1 and KDM5B can bind to the promoter region of DPP4 across cell lines (Fig. 5F and Additional file 2: Fig. S6). Additionally, LINC01132 knockdown significantly blocked the interaction between NRF1 and DPP4 (Fig. 5G). However, the effects were not observed for KDM5B. These results indicated that LINC01132 acts as a scaffold in the interaction between NRF1 and DPP4.

### LINC01132 improves the response to anti-PD1 immunotherapy in HCC

**Fig. 6:**
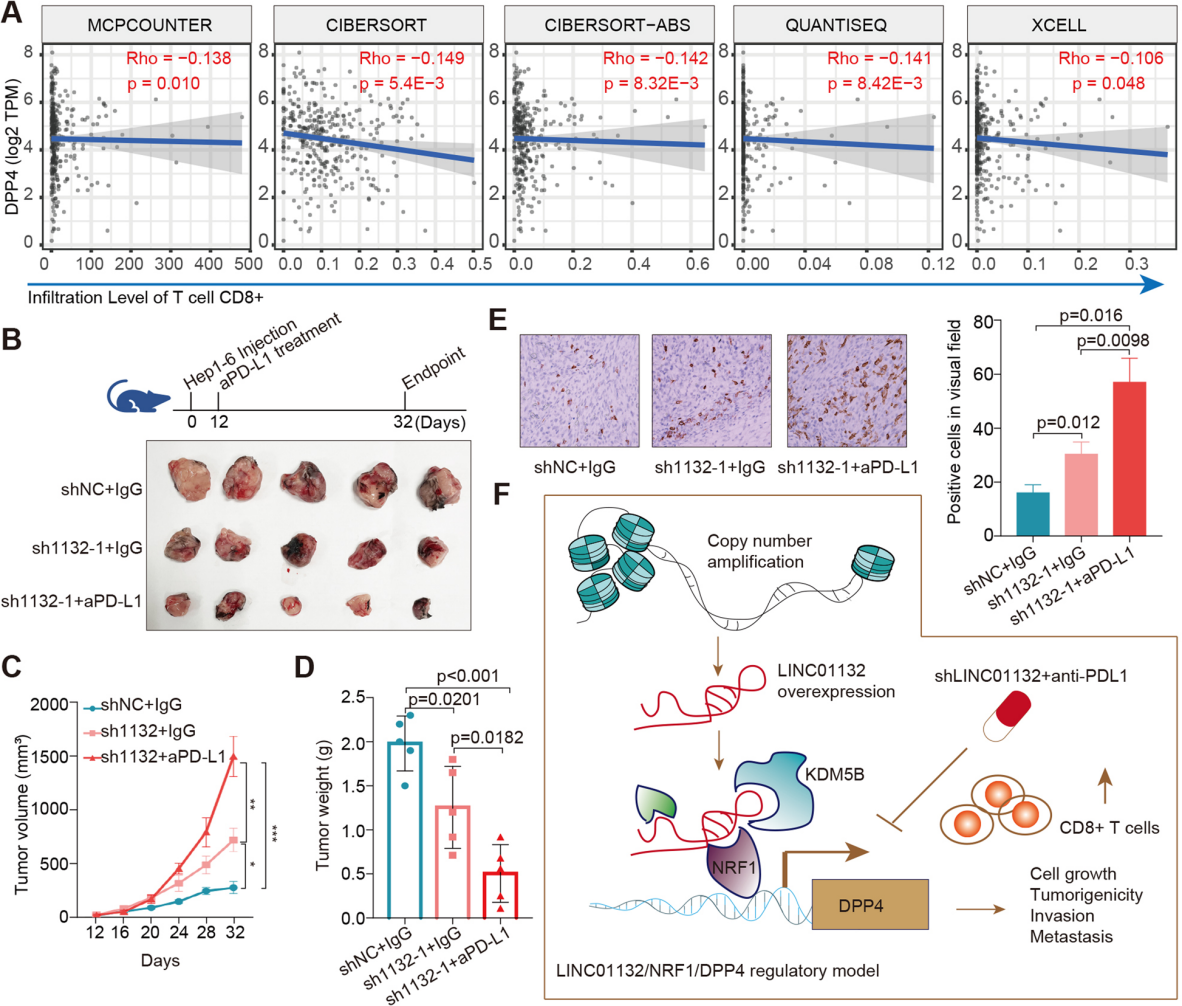
SiLINC01132 improves the response to anti-PDL1 immunotherapy in a subgroup of HCC. **A** Scatter plots showing the correlation between LINC01132 expression and CD8+ T cells infiltrations. **B** Mice were orthotopically xenografted with Hep1–6 injection and treated with anti-PDL1 or IgG or siLINC01132 plus anti-PDL1. Bottom panel showing representative images of tumors treated with IgG + shLINC01132 or anti-PDL1 + shLINC01132. **C**-**D** The tumor volume and weight of mice treated with IgG + shLINC01132 or anti-PDL1 + shLINC01132. **C** for tumor volume and **D** for tumor weight. **E** IHC staining of CD8A and the number of CD8A positive cells. **F** The mechanistic scheme of lncRNA LINC01132/NRF1/DPP4 axis in HCC

Previous studies have identified DPP4 decreased chemokines and other immune molecules [39]. Our data revealed that LINC01132 overexpression was significantly associated with decreased immune pathway activity. We also identified a negative relationship between DPP4 and CD8+ T cell infiltration levels in HCC tissues (Fig. 6A). Combination therapy with anti-PDL1 blockade immunotherapy and other therapies have been shown to improve the efficacy of the tumor-specific T-cell response [40, 41]. Based on the above results of LINC01132, we predicted that LINC01132 knockdown could enhance lymphocyte trafficking and improve tumor responses to PDL1 blockage in HCC. Thus, we investigated the combination therapy of LINC01132 knockdown and PDL1 inhibitor in the Hep1–6-shLINC01132 tumor model (Fig. 6B).

We found that shLINC01132 therapy resulted in delayed tumor growth, smaller tumor volume and weight (Fig. 6B-D). Importantly, much clearer tumor regression was observed in the group treated with shLINC01132 plus anti-PDL1 (Fig. 6B-D). The number of CD8+ T cells was significantly increased in the tumors of mice treated with shLINC01132 compared with control (Fig. 6E, *p* = 0.012). Moreover, the increase was even higher in the tumor of mice treated with the shLINC01132 and PDL1 blockage (Fig. 6E, *p* = 0.0098). Thus, the above results further indicated that LINC01132/NRF1/DPP4 axis is involved in the immunosuppression of HCC (Fig. 6F) and suggested that knockdown LINC01132 could improve the efficacy of PDL1 blockage immunotherapy.

## Discussion

Rapid progresses in high throughput sequencing technologies have successfully identified a large number of lncRNAs. Moreover, emerging evidence has indicated that lncRNAs are expressed in a cell type-specific or tissue-specific patterns [42, 43], suggesting important roles in diverse biological processes. Expression perturbation of lncRNAs has been observed in various cancer types. By integration analysis of genome-wide lncRNA expression and genetic alterations, we revealed that LINC01132 is up-regulated in HCC tissues and that its high expression might be driven by copy number amplification. Moreover, overexpression of LINC01132 was associated with poor prognosis of HCC patients.

LINC01132 promoted cell growth, proliferation, invasion and metastasis in vitro and in vivo. Functional analysis revealed that LINC01132 physically interacted with NRF1 and KDM5B and promoted the expression of DPP4. To date, expression perturbation of LINC01132 has only been associated with oncogenic activities in ovarian cancer by regulating miR-431-5p/SOX9 axis [44] and involved in hypoxia regulation in glioblastoma [45]. Therefore, the roles of LINC01132 in the regulation of NRF1/DPP4 axis in HCC are here first described. These results suggested that the same lncRNA can play diverse functions in regulating various pathways in different tumor context.

CD26/DPP4 is a membrane-bound protein, and its higher expression has been found in a wide variety of tumor pathologies [36]. CD26 expression was significantly increased in tumor HCC specimens and was associated with larger tumor size [46]. CD26 has been proven as a pro-oncogenic gene in HCC and a potential therapeutic target. We also found that DPP4 interacts with number of cancer-related genes (Additional file 2: Fig. S7). Moreover, DPP4 inhibition improves antitumor effect of PD1 in HCC by enhancing CD8+ T cell infiltration [47]. It is increasingly clear that there are widespread changes in lncRNA expressions during the immune response. Numerous lncRNAs, such as NEAT1, UCA1, MIR22HG, and LINK-A, have involved in immune regulation in cancer [48–50]. Here, we demonstrated the inhibition of LINC01132 can achieve the similar antitumor effects. Altogether we expose the critical role of LINC01132/NRF1/DPP4 promoting the development of HCC.

## Conclusions

In conclusion, our results demonstrated that LINC01132 may act as an onco-lncRNA and overexpression of LINC01132 promoted HCC development via the NRF1/DPP4 signaling axis. Contrastingly, LINC01132 silencing may be a novel synergistic strategy to improve the efficacy of PDL1 inhibitor therapy in a subgroup of ICB resistant HCC patients.

## Supplementary Information

**Additional file 1: Table S1.** Primers, probes and siRNA/shRNA used in this study. **Table S2.** Antibodies for western blot and IHC and in vivo used in this study. **Table S3.** Differentially expressed lncRNAs in HCC.

**Additional file 2: Fig. S1.** Genomic and transcriptome alterations of lncRNAs in cancer. **Fig. S2.** Expression of LINC01132 in HCC cell lines. **Fig. S3.** LINC01132 increases cancer cell growth, proliferation, invasion and metastasis in vitro. **Fig. S4.** PDX models shLINC01132. **Fig. S5.** Expression and clinical association of NRF1 and KDM5B. **Fig. S6.** Integrative Genomics Viewer of KDM5B binding around the TSS of DPP4 in HCC cell lines. **Fig. S7.** Interactions of DPP4 in protein-protein interaction networks.

## Data Availability

All data generated or analysed during this study are included in this published article. The gene expression profiles and clinical data can be found at the GDC portal and GEO (https://www.ncbi.nlm.nih.gov/geo/). Software and resources used for the analyses are described in each method section.

## Abbreviations

DPP4: Dipeptidyl Peptidase 4
GSEA: Gene Set Enrichment Analysis
LncRNA: Long non-coding RNAs
HCC: Hepatocellular carcinoma
KDM5B: Lysine Demethylase 5B
LINC01132: Long Intergenic Non-Protein Coding RNA 1132
NRF1: Nuclear Respiratory Factor 1
PDX: Patient-derived tumor xenograft mouse model
shRNA: Short hairepin RNA or small hairpin RNA
SOX9: SRY-Box Transcription Factor 9

## References

[1] Sung H, Ferlay J, Siegel RL, Laversanne M, Soerjomataram I, Jemal A, Bray F (2021). Global Cancer statistics 2020: GLOBOCAN estimates of incidence and mortality worldwide for 36 cancers in 185 countries. CA Cancer J Clin.

[2] Maluccio M, Covey A (2012). Recent progress in understanding, diagnosing, and treating hepatocellular carcinoma. CA Cancer J Clin.

[3] Ozer Etik D, Suna N, Boyacioglu AS (2017). Management of Hepatocellular Carcinoma: prevention, surveillance, diagnosis, and staging. Exp Clin Transplant.

[4] Rebouissou S, Nault JC (2020). Advances in molecular classification and precision oncology in hepatocellular carcinoma. J Hepatol.

[5] Bang H, Ha SY, Hwang SH, Park CK (2015). Expression of PEG10 is associated with poor survival and tumor recurrence in hepatocellular carcinoma. Cancer Res Treat.

[6] Ke AW, Shi GM, Zhou J, Wu FZ, Ding ZB, Hu MY, Xu Y, Song ZJ, Wang ZJ, Wu JC (2009). Role of overexpression of CD151 and/or c-met in predicting prognosis of hepatocellular carcinoma. Hepatology.

[7] Li Y, McGrail DJ, Xu J, Li J, Liu NN, Sun M, Lin R, Pancsa R, Zhang J, Lee JS (2019). MERIT: systematic analysis and characterization of mutational effect on RNA Interactome topology. Hepatology.

[8] Frankish A, Diekhans M, Jungreis I, Lagarde J, Loveland JE, Mudge JM, Sisu C, Wright JC, Armstrong J, Barnes I (2021). Gencode 2021. Nucleic Acids Res.

[9] Goodall GJ, Wickramasinghe VO (2021). RNA in cancer. Nat Rev Cancer.

[10] Peng WX, Koirala P, Mo YY (2017). LncRNA-mediated regulation of cell signaling in cancer. Oncogene.

[11] Huang Z, Zhou JK, Peng Y, He W, Huang C (2020). The role of long noncoding RNAs in hepatocellular carcinoma. Mol Cancer.

[12] Li Z, Zhang J, Liu X, Li S, Wang Q, Di C, Hu Z, Yu T, Ding J, Li J (2018). The LINC01138 drives malignancies via activating arginine methyltransferase 5 in hepatocellular carcinoma. Nat Commun.

[13] Zhang J, Li S, Zhang L, Xu J, Song M, Shao T, Huang Z, Li Y (2020). RBP EIF2S2 promotes tumorigenesis and progression by regulating MYC-mediated inhibition via FHIT-related enhancers. Mol Ther.

[14] Ding J, Zhao J, Huan L, Liu Y, Qiao Y, Wang Z, Chen Z, Huang S, Zhao Y, He X (2020). Inflammation-induced long intergenic noncoding RNA (LINC00665) increases malignancy through activating the double-stranded RNA-activated protein kinase/nuclear factor kappa B pathway in hepatocellular carcinoma. Hepatology.

[15] Schreiber RD, Old LJ, Smyth MJ (2011). Cancer immunoediting: integrating immunity's roles in cancer suppression and promotion. Science.

[16] Pinter M, Scheiner B, Peck-Radosavljevic M (2021). Immunotherapy for advanced hepatocellular carcinoma: a focus on special subgroups. Gut.

[17] Zongyi Y, Xiaowu L (2020). Immunotherapy for hepatocellular carcinoma. Cancer Lett.

[18] Llovet JM, Castet F, Heikenwalder M, Maini MK, Mazzaferro V, Pinato DJ, Pikarsky E, Zhu AX, Finn RS (2022). Immunotherapies for hepatocellular carcinoma. Nat Rev Clin Oncol.

[19] Li G, Kryczek I, Nam J, Li X, Li S, Li J, Wei S, Grove S, Vatan L, Zhou J (2021). LIMIT is an immunogenic lncRNA in cancer immunity and immunotherapy. Nat Cell Biol.

[20] Huang D, Chen J, Yang L, Ouyang Q, Li J, Lao L, Zhao J, Liu J, Lu Y, Xing Y (2018). NKILA lncRNA promotes tumor immune evasion by sensitizing T cells to activation-induced cell death. Nat Immunol.

[21] Li Y, Jiang T, Zhou W, Li J, Li X, Wang Q, Jin X, Yin J, Chen L, Zhang Y (2020). Pan-cancer characterization of immune-related lncRNAs identifies potential oncogenic biomarkers. Nat Commun.

[22] Kim D, Paggi JM, Park C, Bennett C, Salzberg SL (2019). Graph-based genome alignment and genotyping with HISAT2 and HISAT-genotype. Nat Biotechnol.

[23] Pertea M, Pertea GM, Antonescu CM, Chang TC, Mendell JT, Salzberg SL (2015). StringTie enables improved reconstruction of a transcriptome from RNA-seq reads. Nat Biotechnol.

[24] Pertea M, Kim D, Pertea GM, Leek JT, Salzberg SL (2016). Transcript-level expression analysis of RNA-seq experiments with HISAT, StringTie and Ballgown. Nat Protoc.

[25] Cancer Genome Atlas Research Network (2017). Electronic address wbe, Cancer genome atlas research N: comprehensive and integrative genomic characterization of hepatocellular carcinoma. Cell.

[26] Yoo S, Wang W, Wang Q, Fiel MI, Lee E, Hiotis SP, Zhu J (2017). A pilot systematic genomic comparison of recurrence risks of hepatitis B virus-associated hepatocellular carcinoma with low- and high-degree liver fibrosis. BMC Med.

[27] Subramanian A, Tamayo P, Mootha VK, Mukherjee S, Ebert BL, Gillette MA, Paulovich A, Pomeroy SL, Golub TR, Lander ES, Mesirov JP (2005). Gene set enrichment analysis: a knowledge-based approach for interpreting genome-wide expression profiles. Proc Natl Acad Sci U S A.

[28] Liberzon A, Birger C, Thorvaldsdottir H, Ghandi M, Mesirov JP, Tamayo P (2015). The molecular signatures database (MSigDB) hallmark gene set collection. Cell Syst.

[29] Zhou KR, Liu S, Sun WJ, Zheng LL, Zhou H, Yang JH, Qu LH (2017). ChIPBase v2.0: decoding transcriptional regulatory networks of non-coding RNAs and protein-coding genes from ChIP-seq data. Nucleic Acids Res.

[30] Luo Z, Cao P (2019). Long noncoding RNA PVT1 promotes hepatoblastoma cell proliferation through activating STAT3. Cancer Manag Res.

[31] Hong F, Gao Y, Li Y, Zheng L, Xu F, Li X (2020). Inhibition of HIF1A-AS1 promoted starvation-induced hepatocellular carcinoma cell apoptosis by reducing HIF-1alpha/mTOR-mediated autophagy. World J Surg Oncol.

[32] Huang X, Gao Y, Qin J, Lu S (2018). lncRNA MIAT promotes proliferation and invasion of HCC cells via sponging miR-214. Am J Physiol Gastrointest Liver Physiol.

[33] Chen MM, Li J, Wang Y, Akbani R, Lu Y, Mills GB, Liang H (2019). TCPA v3.0: an integrative platform to explore the Pan-Cancer analysis of functional proteomic data. Mol Cell Proteomics.

[34] Steen CB, Luca BA, Esfahani MS, Azizi A, Sworder BJ, Nabet BY, Kurtz DM, Liu CL, Khameneh F, Advani RH (2021). The landscape of tumor cell states and ecosystems in diffuse large B cell lymphoma. Cancer Cell.

[35] Aguilar-Melero P, Prieto-Alamo MJ, Jurado J, Holmgren A, Pueyo C (2013). Proteomics in HepG2 hepatocarcinoma cells with stably silenced expression of PRDX1. J Proteome.

[36] Enz N, Vliegen G, De Meester I, Jungraithmayr W (2019). CD26/DPP4 - a potential biomarker and target for cancer therapy. Pharmacol Ther.

[37] Ji P, Wang H, Cheng Y, Liang S (2021). Prognostic prediction and gene regulation network of EIF2S2 in hepatocellular carcinoma based on data mining. J Gastrointest Oncol.

[38] Zhang J, Li S, Zhang L, Xu J, Song M, Shao T, Huang Z, Li Y (2021). RBP EIF2S2 promotes tumorigenesis and progression by regulating MYC-mediated inhibition via FHIT-related enhancers. Mol Ther.

[39] Hollande C, Boussier J, Ziai J, Nozawa T, Bondet V, Phung W, Lu B, Duffy D, Paradis V, Mallet V (2019). Inhibition of the dipeptidyl peptidase DPP4 (CD26) reveals IL-33-dependent eosinophil-mediated control of tumor growth. Nat Immunol.

[40] Hayashi H, Nakagawa K (2020). Combination therapy with PD-1 or PD-L1 inhibitors for cancer. Int J Clin Oncol.

[41] Xu J, Shao T, Song M, Xie Y, Zhou J, Yin J, Ding N, Zou H, Li Y, Zhang J (2020). MIR22HG acts as a tumor suppressor via TGFbeta/SMAD signaling and facilitates immunotherapy in colorectal cancer. Mol Cancer.

[42] Lv D, Xu K, Jin X, Li J, Shi Y, Zhang M, Jin X, Li Y, Xu J, Li X (2020). LncSpA: LncRNA spatial atlas of expression across normal and cancer tissues. Cancer Res.

[43] Xu K, Cai Y, Zhang M, Zou H, Chang Z, Li D, Bai J, Xu J, Li Y (2021). Pan-cancer characterization of expression and clinical relevance of m (6) A-related tissue-elevated long non-coding RNAs. Mol Cancer.

[44] Zhu W, Xiao X, Chen J. Silencing of the long noncoding RNA LINC01132 alleviates the oncogenicity of epithelial ovarian cancer by regulating the microRNA4315p/SOX9 axis. Int J Mol Med. 2021;48. 10.3892/ijmm.2021.4984 PMC8219520 34132375

[45] Bao L, Chen Y, Lai HT, Wu SY, Wang JE, Hatanpaa KJ, Raisanen JM, Fontenot M, Lega B, Chiang CM (2018). Methylation of hypoxia-inducible factor (HIF)-1alpha by G9a/GLP inhibits HIF-1 transcriptional activity and cell migration. Nucleic Acids Res.

[46] Kawaguchi T, Kodama T, Hikita H, Makino Y, Saito Y, Tanaka S, Shimizu S, Sakamori R, Miyagi T, Wada H (2015). Synthetic lethal interaction of combined CD26 and Bcl-xL inhibition is a powerful anticancer therapy against hepatocellular carcinoma. Hepatol Res.

[47] Huang XY, Zhang PF, Wei CY, Peng R, Lu JC, Gao C, Cai JB, Yang X, Fan J, Ke AW (2020). Circular RNA circMET drives immunosuppression and anti-PD1 therapy resistance in hepatocellular carcinoma via the miR-30-5p/snail/DPP4 axis. Mol Cancer.

[48] Heward JA, Lindsay MA (2014). Long non-coding RNAs in the regulation of the immune response. Trends Immunol.

[49] Wang CJ, Zhu CC, Xu J, Wang M, Zhao WY, Liu Q, Zhao G, Zhang ZZ (2019). The lncRNA UCA1 promotes proliferation, migration, immune escape and inhibits apoptosis in gastric cancer by sponging anti-tumor miRNAs. Mol Cancer.

[50] Zhang M, Zheng Y, Sun Y, Li S, Chen L, Jin X, Hou X, Liu X, Chen Q, Li J (2019). Knockdown of NEAT1 induces tolerogenic phenotype in dendritic cells by inhibiting activation of NLRP3 inflammasome. Theranostics.

