# Supplementary material for: Long noncoding RNA LINC01132 enhances immunosuppression and therapy resistance via NRF1/DPP4 axis in hepatocellular carcinoma
Source: J Exp Clin Cancer Res. 2022 Sep 8;41:270. doi: 10.1186/s13046-022-02478-z (PMC9454129; doi:10.1186/s13046-022-02478-z)
Supplement: Supplementary file 1 — Additional file 1: Table S1. Primers, probes and siRNA/shRNA used in this study. Table S2. Antibodies for western blot and IHC and in vivo used in this study. Table S3. Differentially expressed lncRNAs in HCC. [file 13046_2022_2478_MOESM1_ESM.doc]

**Table S1. Primers, probes and siRNA/shRNA used in this study.**

| **Names** | **Forward-primer** | **Reverse-primer** |
| --- | --- | --- |
| LINC01132-qPCR | CTAGCCAAAGGCTGGCTTCT | AGCTCTCACACTTGACGGTG |
| β-actin-qPCR | AGTGTGACGTGGACATCCGCAAAG | ATCCACATCTGCTGGAAGGTGGAC |
| LINC01132-ORF | CGTAAATTTGTTTGTGTCTC | TTACGTTGTCCACATAAACA |
| LINC01132-northern | TCTTCCTGCTAGCCAAAGGC | CCTGTTTTGGAATGCGGTGG TAATACGACTCACTATAG |
| 5’RACE | GCGGCAGGGTGTGTATTTTC |  |
| 3’RACE | GATGAGCCCCTGAAAGGCAT |
| si-LINC01132-1 | GTGACTTAAGGCTCATGTG |
| si-LINC01132-2 | AGGAGATAAAAATTTTAAATTAC |
| si-LINC01132-3 | TTCTGTTTTTTGTTTTTTTAAGA |
| si-NC | CACGATAAGACAATGTAT TT |

**Table S2. Antibodies for western blot and IHC and in vivo used in this study.**

| **Name** | **Company** | **Catalog number** |
| --- | --- | --- |
| NRF1 | Cell Signaling Technology | 46743 |
| KDM5B | Thermo Fisher | MA5-24626 |
| DPP4 | Cell Signaling Technology | 67138 |
| GAPDH | Cell Signaling Technology | 5174 |
| Cd8a | Thermo Fisher | 14-0081-82 |
| Mouse CD274 | Bio X Cell | BE0101 |
| Mouse IgG2b | Bio X Cell | BP0086 |

**Table S3. Differentially expressed lncRNAs in HCC.**

| Ensemble IDs | LncRNA names | lincRNA | Fold changes | P-values |
| --- | --- | --- | --- | --- |
| ENSG00000198468 | FLVCR1-AS1 | lincRNA | 4.904573 | 0.015860919 |
| ENSG00000227630 | LINC01132 | lincRNA | 2.308049 | 0.025244106 |
| ENSG00000163364 | LINC01116 | lincRNA | 23.54678 | 0.014334924 |
| ENSG00000203645 | LINC00501 | lincRNA | 15.531119 | 0.025222573 |
| ENSG00000226673 | LINC01108 | lincRNA | 4.999413 | 0.014334924 |
| ENSG00000230658 | KLHL7-AS1 | lincRNA | 4.17708 | 0.018572051 |
| ENSG00000214293 | APTR | lincRNA | 2.23008 | 0.004096213 |
| ENSG00000249859 | PVT1 | lincRNA | 3.62284 | 0.031420179 |
| ENSG00000232850 | PTGES2-AS1 | lincRNA | 4.105672 | 0.000714456 |
| ENSG00000225383 | SFTA1P | lincRNA | 43.613113 | 0.000609111 |
| ENSG00000237523 | LINC00857 | lincRNA | 2.477707 | 0.007303326 |
| ENSG00000248265 | FLJ12825 | lincRNA | 5.016579 | 0.038222852 |
| ENSG00000278916 | CEP83-AS1 | lincRNA | 2.85539 | 0.031420179 |
| ENSG00000256128 | LINC00944 | lincRNA | 13.226557 | 0.005135132 |
| ENSG00000188825 | LINC00910 | lincRNA | 2.336015 | 0.009557831 |
| ENSG00000187013 | C17orf82 | lincRNA | 5.697434 | 0.001360045 |
| ENSG00000185168 | LINC00482 | lincRNA | 4.08658 | 0.038707629 |
| ENSG00000268658 | LINC00664 | lincRNA | 14.135009 | 0.038268815 |
| ENSG00000269220 | LINC00528 | lincRNA | 4.198247 | 0.005493371 |
| ENSG00000236499 | LINC00896 | lincRNA | 3.079046 | 0.02058991 |
| ENSG00000203650 | LINC01285 | lincRNA | 6.228646 | 0.033197006 |
| ENSG00000213468 | FIRRE | lincRNA | 4.785397 | 0.021831749 |
| ENSG00000198468 | FLVCR1-AS1 | lincRNA | 4.904573 | 0.015860919 |
| ENSG00000227630 | LINC01132 | lincRNA | 2.308049 | 0.025244106 |
| ENSG00000163364 | LINC01116 | lincRNA | 23.54678 | 0.014334924 |
| ENSG00000203645 | LINC00501 | lincRNA | 15.531119 | 0.025222573 |
| ENSG00000226673 | LINC01108 | lincRNA | 4.999413 | 0.014334924 |
| ENSG00000230658 | KLHL7-AS1 | lincRNA | 4.17708 | 0.018572051 |
| ENSG00000281664 | LINC00538 | lincRNA | 0.45989489 | 0.009722284 |
| ENSG00000205837 | LINC00487 | lincRNA | 0.42021749 | 0.038707629 |
| ENSG00000228784 | LINC00954 | lincRNA | 0.31677044 | 0.018439495 |
| ENSG00000237803 | LINC00211 | lincRNA | 0.22417562 | 0.013473345 |
| ENSG00000233723 | LINC01122 | lincRNA | 0.10133806 | 0.029254566 |
| ENSG00000224957 | LINC01266 | lincRNA | 0.09624681 | 0.017025101 |
| ENSG00000241163 | LINC00877 | lincRNA | 0.39851226 | 0.011838928 |
| ENSG00000271856 | LINC01215 | lincRNA | 0.38089672 | 0.039392109 |
| ENSG00000224652 | LINC00885 | lincRNA | 0.30233069 | 0.012128869 |
| ENSG00000249173 | LINC01093 | lincRNA | 0.21332249 | 0.002159244 |
| ENSG00000250056 | LINC01018 | lincRNA | 0.46840032 | 0.031420179 |
| ENSG00000233237 | LINC00472 | lincRNA | 0.49363027 | 0.012399778 |
| ENSG00000274956 | UG0898H09 | lincRNA | 0.28153748 | 0.008527792 |
| ENSG00000245164 | LINC00861 | lincRNA | 0.39411123 | 0.002159244 |
| ENSG00000250400 | LINC00977 | lincRNA | 0.15184494 | 0.012726493 |
| ENSG00000231298 | LINC00704 | lincRNA | 0.23940186 | 0.003682797 |
| ENSG00000238266 | LINC00707 | lincRNA | 0.07754244 | 0.000180839 |
| ENSG00000237949 | LINC00844 | lincRNA | 0.27345854 | 0.003760918 |
| ENSG00000226051 | ZNF503-AS1 | lincRNA | 0.35447783 | 0.000301659 |
| ENSG00000232229 | LINC00865 | lincRNA | 0.45026857 | 0.038707629 |
| ENSG00000240707 | LINC01168 | lincRNA | 0.14617322 | 0.006087404 |
| ENSG00000205866 | FAM99A | lincRNA | 0.38887349 | 0.012399778 |
| ENSG00000215483 | LINC00598 | lincRNA | 0.07527951 | 0.025244106 |
| ENSG00000258777 | HIF1A-AS1 | lincRNA | 0 | 0.029867793 |
| ENSG00000260551 | PWRN2 | lincRNA | 0.24810737 | 0.041662214 |
| ENSG00000259905 | PWRN1 | lincRNA | 0.15670335 | 0.005066612 |
| ENSG00000248441 | LINC01197 | lincRNA | 0.40874152 | 0.015860919 |
| ENSG00000268388 | FENDRR | lincRNA | 0.11301816 | 0.000425609 |
| ENSG00000267586 | LINC00907 | lincRNA | 0.14974585 | 0.001524172 |
