# Supplementary material for: Long noncoding RNA LINC01132 enhances immunosuppression and therapy resistance via NRF1/DPP4 axis in hepatocellular carcinoma
Source: J Exp Clin Cancer Res. 2022 Sep 8;41:270. doi: 10.1186/s13046-022-02478-z (PMC9454129; doi:10.1186/s13046-022-02478-z)
Supplement: Supplementary file 2 — Additional file 2: Fig. S1. Genomic and transcriptome alterations of lncRNAs in cancer. Fig. S2. Expression of LINC01132 in HCC cell lines. Fig. S3. LINC01132 increases cancer cell growth, proliferation, invasion and metastasis in vitro. Fig. S4. PDX models shLINC01132. Fig. S5. Expression and clinical association of NRF1 and KDM5B. Fig. S6. Integrative Genomics Viewer of KDM5B binding around the TSS of DPP4 in HCC cell lines. Fig. S7. Interactions of DPP4 in protein-protein interaction networks. [file 13046_2022_2478_MOESM2_ESM.docx]

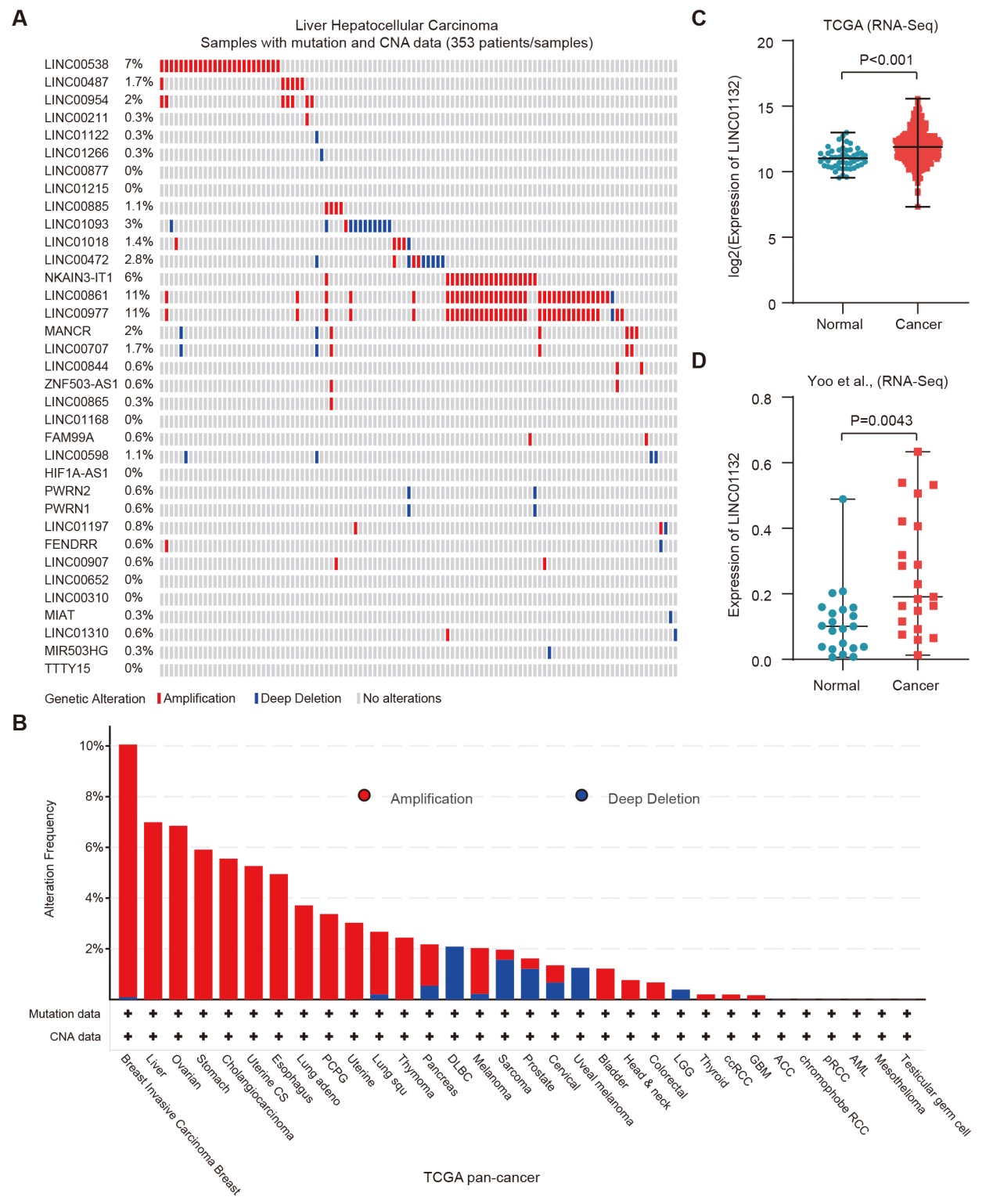


**Fig. S1. Genomic and transcriptome alterations of lncRNAs in cancer.** **A,** Waterfall plot showing the genetic alterations of down-regulated lncRNAs in HCC. **B,** Frequency of copy number alteration for LINC01132 across cancer types. Red for copy number amplification and blue for deep deletion. **C,** Boxplot showing the expressions of LINC01132 in tumor and normal samples of TCGA cohort. **D,** Boxplot showing the expressions of LINC01132 in tumor and normal samples of Yoo et al. cohort.


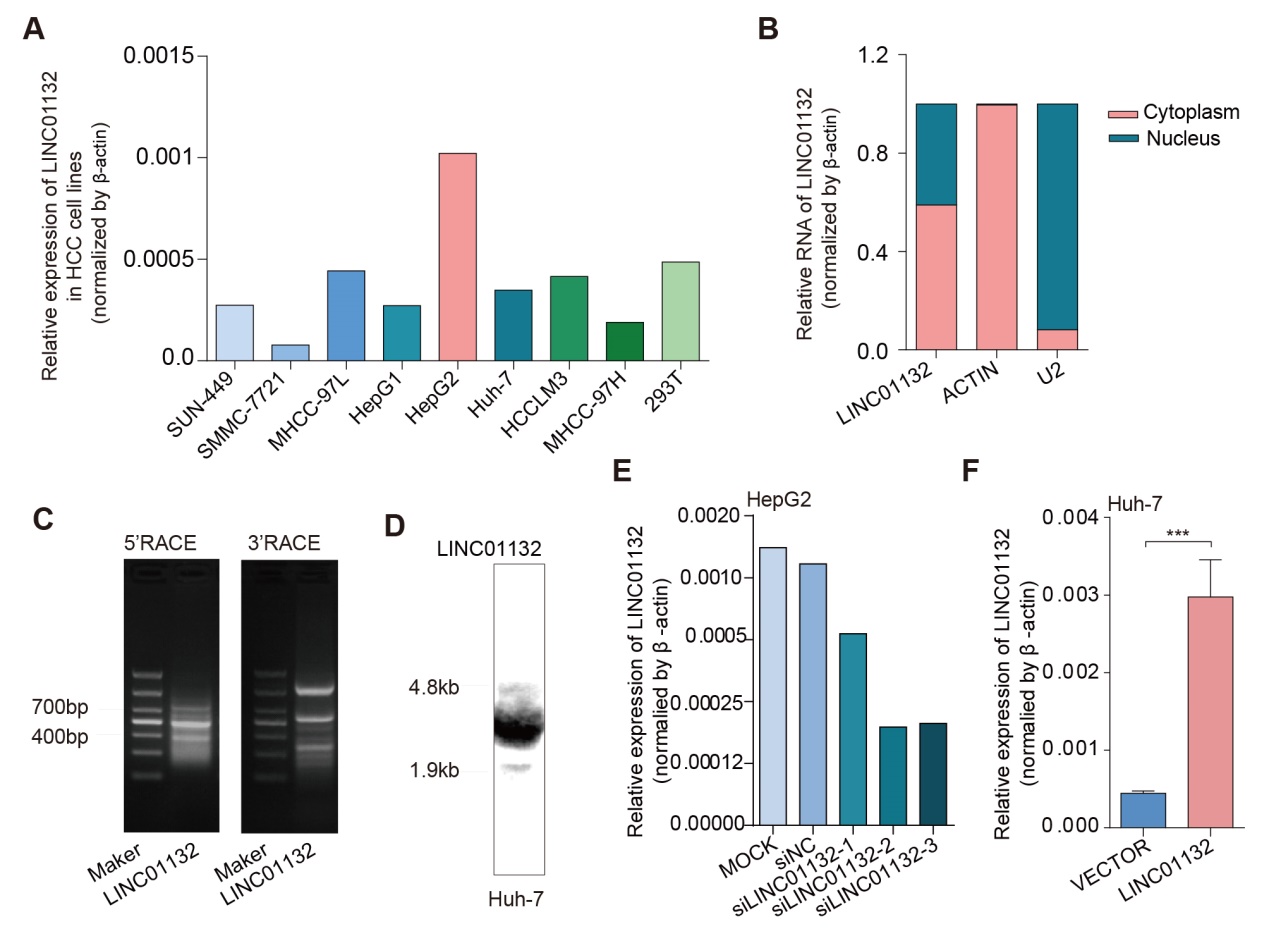


**Fig. S2. Expression of LINC01132 in HCC cell lines. A,** Relative expression of LINC01132 across cancer cell lines. **B,** Relative expression of LINC01132 in cytoplasm and nucleus. **C,** Rapid-amplification of cDNA ends (RACE) for LINC01132. **D,** Northern blot for LINC01132. **E,** Relative expression of LINC01132 in HepG2 with different experimental conditions. **F,** Relative expression of LINC01132 in overexpressing Huh-7 cell line.


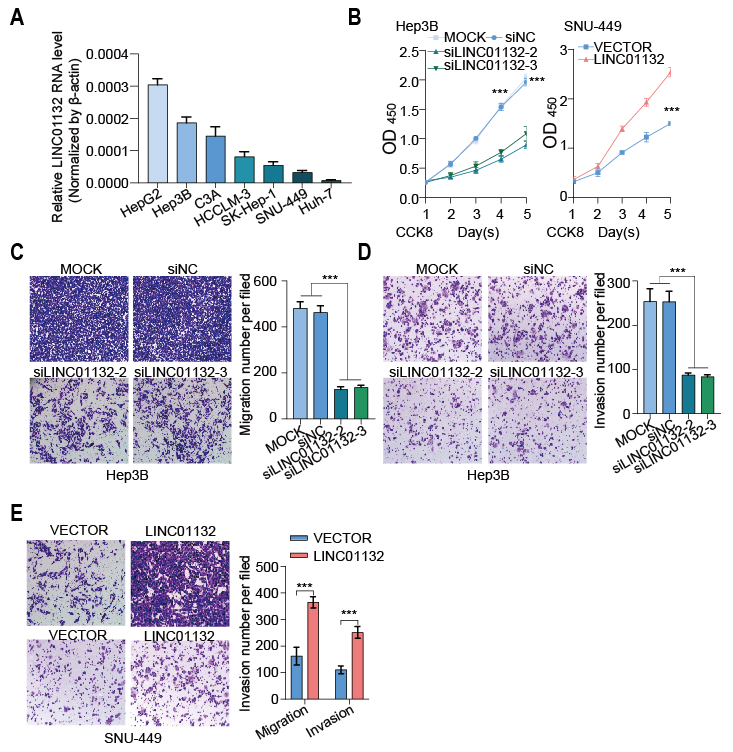


**Fig. S3. LINC01132 increases cancer cell growth, proliferation, invasion and metastasis in vitro.** **A,** Relative expression of LINC01132 across cancer cell lines. **B,** Cell Counting Kit-8 (CCK-8) assays showing the growth of HCC cells treated with siLINC01132 or overexpression of LINC01132. **C-E,** Transwell migration and invasion assays in HCC cell lines treated with siLINC01132 or overexpression of LINC01132. **C** for migration treated with siLINC01132, **D** for invasion treated with siLINC01132 and **E** for invasion treated with overexpression of LINC01132.


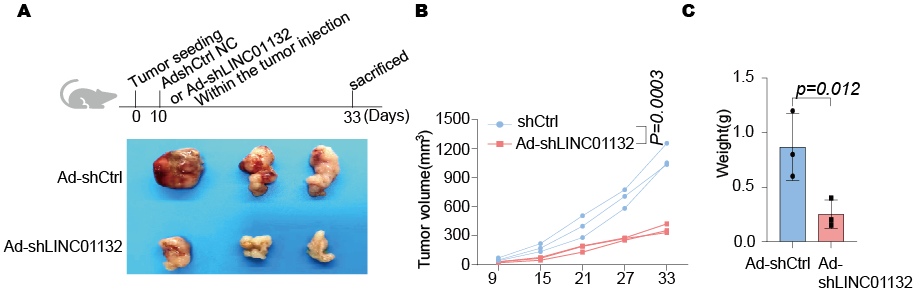


**Fig. S4. PDX models shLINC01132.** **A**, Adenovirus vector PDX model. **B**, The lines showing the tumor volumes of PDXs. **C**, The histogram showing the tumor weights of PDXs.


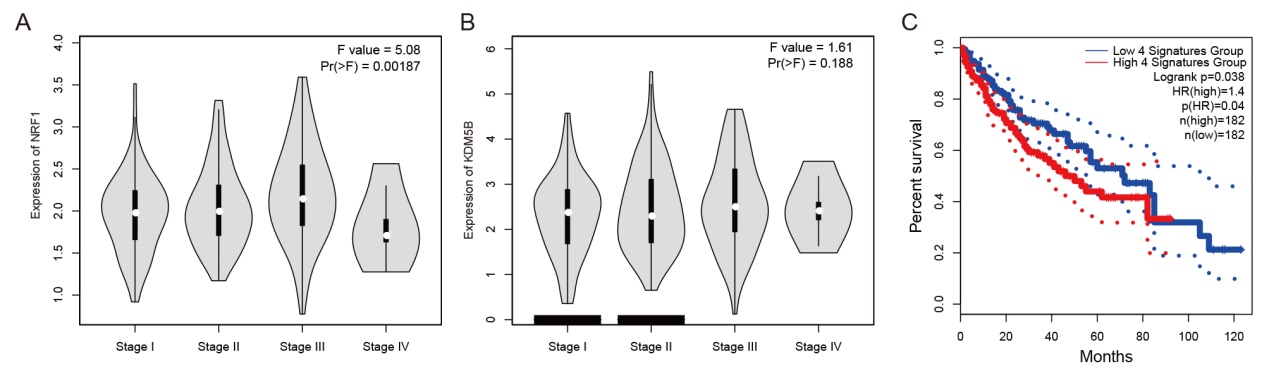


**Fig. S5. Expression and clinical association of NRF1 and KDM5B. A,** Expression of NRF1 in HCC patients with different stages. **B,** Expression of KDM5B in HCC patients with different stages. **C,** Kaplan-Meier survival analysis of HCC patients stratified by the LINC01132/NRF1/KDM5B/DPP4 signature expression level.


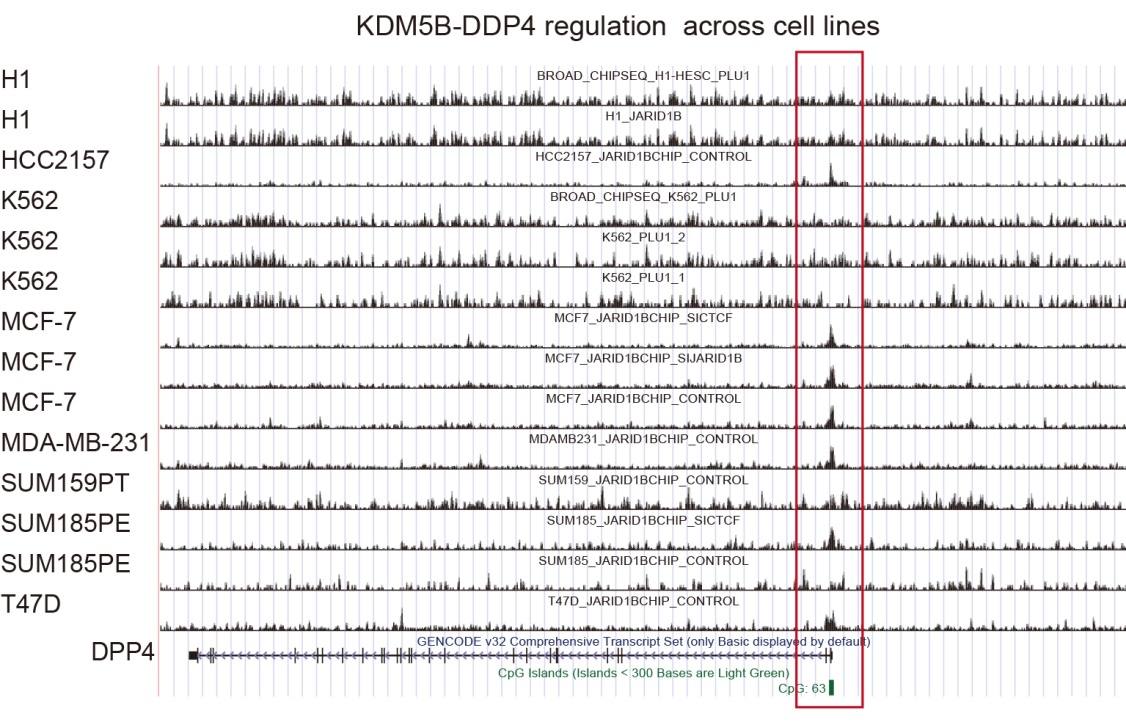


**Fig. S6. Integrative Genomics Viewer of KDM5B binding around the TSS of DPP4 in HCC cell lines.**


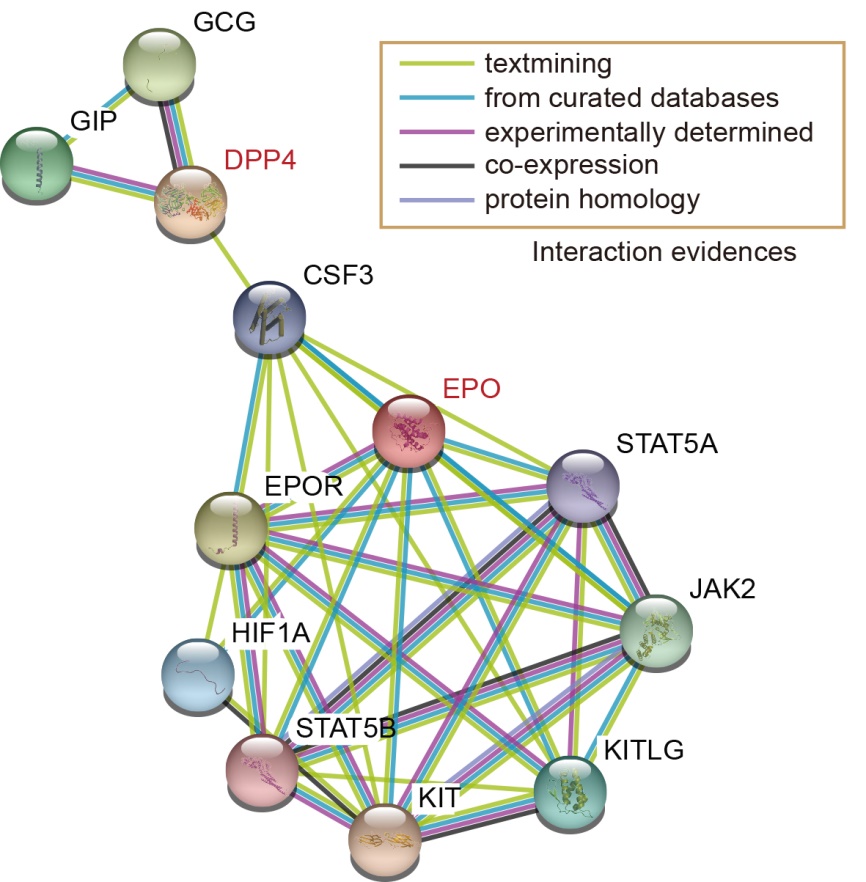


**Fig. S7. Interactions of DPP4 in protein-protein interaction networks.**
